# Platelet-Based Markers Associated With Vascular Access Dysfunction in Hemodialysis Patients

**DOI:** 10.7759/cureus.105507

**Published:** 2026-03-19

**Authors:** Diana D Nenova, Yanko G Yankov, Gergana M Chausheva, Lyuben L Stoev

**Affiliations:** 1 Second Department of Internal Disease, Medical University "Prof. Dr. Paraskev Stoyanov", Varna, BGR; 2 Clinic of Nephrology and Dialysis, University Hospital "St. Marina", Varna, BGR; 3 Clinic of Maxillofacial Surgery, University Hospital "St. Marina", Varna, BGR; 4 Department of General and Operative Surgery, Medical University "Prof. Dr. Paraskev Stoyanov", Varna, BGR; 5 Department of Clinical Laboratory, Medical University "Prof. Dr. Paraskev Stoyanov", Varna, BGR; 6 Department of Central Clinical Laboratory, University Hospital "St. Marina", Varna, BGR; 7 Department of General and Clinical Pathology, Forensic Medicine and Deontology, Medical University "Prof. Dr. Paraskev Stoyanov", Varna, BGR; 8 Clinic of General and Clinical Pathology, University Hospital "St. Marina", Varna, BGR

**Keywords:** activation, complication, dialysis, discriminatory, dysfunction, mean platelet volume, outcome, platelet count, thrombosis, vascular access

## Abstract

Introduction

Vascular access (VA) dysfunction is a major determinant of inadequate dialysis delivery and clinical complications in hemodialysis (HD) patients. Mean platelet volume (MPV), a marker of platelet activation, has emerged as a potential discriminatory marker for thrombotic events and VA complications. The aim of this study was to evaluate the association and discriminatory performance of platelet indices, particularly MPV, in identifying VA dysfunction in patients undergoing chronic HD. Secondary objectives included exploring the relationship of platelet parameters with dialysis adequacy and selected hematologic parameters. By evaluating the discriminatory performance of these routinely available platelet indices, this study also explored their potential clinical relevance as accessible biomarkers that may help identify VA dysfunction in HD patients.

Materials and methods

This retrospective study analyzed 104 HD patients treated between 2017 and 2021. Patients were grouped by VA: arteriovenous fistula (AVF, n=51) or permanent catheter (PC, n=53). MPV and platelet count (PLT) were evaluated against access dysfunction (Qb<250 mL/min), dialysis adequacy indices (single pool (spKt/V), urea reduction ratio (URR%)), and anemia control parameters (hemoglobin (Hgb) levels; erythropoiesis-stimulating agents (ESAs) doses used). Correlation and comparative analyses, effect size estimation, and receiver operating characteristic (ROC) curve analysis were performed.

MPV was higher in PC patients (11.34±1.09 fL) than AVF patients (10.11±1.33 fL; p<0.00001). Higher MPV correlated with lower spKt/V, lower Qb, higher ESA requirements, and lower Hgb in both groups (p<0.001). ROC analysis showed excellent discriminatory performance of MPV for identifying VA dysfunction with an AUC of 0.96 (95% CI: 0.904-1.00, p<0.0001) in AVF and an AUC of 0.956 (95% CI: 0.884-1.00; p< 0.001) in PC, with optimal cut-off values >10.27 fL and >11.5 fL, respectively. PLT showed a statistically significant inverse discriminatory ability AUC of 0.053 (95% CI: 0.010-0.096; p<0.001) in AVF and AUC of 0.086 (95% CI: 0.05-0.167; p<0.001) in PC with poor diagnostic performance.

Discussion

MPV was strongly associated with VA dysfunction in patients undergoing HD. Higher MPV values were linked to reduced dialysis blood flow, lower dialysis adequacy, higher ESA requirements, and lower hemoglobin levels. ROC analysis demonstrated strong discriminatory performance (AUC≈0.96) with clearly identifiable cutoff values across VA types. Although PLT showed a statistical association with VA dysfunction, its inverse discriminatory pattern and lack of a clinically useful threshold limit its practical applicability. The consistent inverse relationship between MPV and PLT supports the potential relevance of platelet activation, rather than platelet quantity, in the pathophysiology of VA dysfunction.

Conclusion

MPV may represent a readily available laboratory parameter associated with VA dysfunction and dialysis performance in HD patients. Given its routine availability as part of the CBC, MPV may have potential clinical relevance for identifying patients with VA impairment. Prospective studies are needed to validate these findings and to clarify the potential role of platelet indices in VA monitoring.

## Introduction

Vascular access (VA) dysfunction remains a major challenge in chronic hemodialysis (HD), frequently leading to thrombosis, stenosis, inadequate dialysis delivery, and increased morbidity. Permanent VA in HD patients is typically achieved via an arteriovenous fistula (AVF) or a permanent catheter (PC), each associated with a distinct risk profile for complications and long-term patency. To meet dialysis goals, the required blood flow should be at least 250 mL/minute, and recent guidelines suggest aiming for over 300 mL/minute during a four-hour session [1-14]. Platelet activation and reactivity are believed to play an important role in the pathophysiology of VA failure; however, reliable and easily measurable biomarkers associated with access dysfunction remain limited [15].

Mean platelet volume (MPV) is a readily available laboratory parameter that is automatically calculated as part of the complete blood count (CBC). It complements the standard quantitative assessment of platelet count (PLT) by providing additional information on platelet size and indirectly reflecting platelet activation and production dynamics [16]. Larger platelets are metabolically and enzymatically more active, and elevated MPV has been associated with increased cardiovascular risk and thrombotic events in various populations [17]. In the context of HD, several studies have suggested a potential association between MPV and VA events. For instance, in a prospective cohort of 153 HD patients, those in the highest MPV quartile had significantly more VA dysfunction events compared with those in the lowest quartile; multivariate analysis confirmed MPV as an independent risk factor (OR 1.52, p<0.001) [15]. Similarly, an increased MPV/PLT ratio has been associated with AVF failure [16].

Given the ease and low cost of obtaining MPV, investigating its potential role as a biomarker associated with VA dysfunction, dialysis adequacy, and anemia management may have important clinical implications. This may be particularly relevant in settings where advanced vascular surveillance techniques, such as routine access flow measurements or Doppler ultrasonography, are not feasible for every dialysis session.

Both increased platelet activation and impaired platelet function have been reported in dialysis patients. Clinically, these abnormalities contribute to the paradoxical coexistence of a prothrombotic tendency and an increased risk of bleeding. The mechanisms behind bleeding in uremia are thought to be multifactorial, but the most significant issues affect primary hemostasis, as disruptions in PLT-PLT and PLT-vessel wall interactions play a key role. Altered platelet function is partly attributed to uremic toxins circulating in the blood. Although platelet function is reduced, disturbances in coagulation and fibrinolysis are less consistent and often suggest a hypercoagulable state, contributing to cardiovascular and thrombotic events, as well as VA dysfunction [18-21].

The aim of this study was to evaluate the association and discriminatory performance of platelet indices, particularly MPV, in identifying VA dysfunction in patients undergoing chronic HD. Secondary objectives included exploring the relationship between platelet parameters, dialysis adequacy, and selected hematologic parameters. By evaluating the discriminatory performance of these routinely available platelet indices, this study also explored their potential clinical relevance as accessible biomarkers that may help identify VA dysfunction in HD patients.

## Materials and methods

Study design

This retrospective observational study was conducted at the Clinic of Nephrology and Dialysis at the University Hospital “St. Marina” in Varna, Bulgaria, covering a five-year period from January 2017 to December 2021. All data were obtained from routine medical records, and no additional procedures were introduced for research purposes. The study adhered to STROBE (STrengthening the Reporting of OBservational studies in Epidemiology) guidelines [22].

Participants and eligibility criteria

A total of 156 chronic HD patients were screened. The inclusion criteria required participants to be at least 18 years old, to have undergone HD for more than six months, and to have minimal residual renal function, defined as a urine output of less than 100 ml per day. Patients also needed to have permanent VA, to have received the same baseline dose of erythropoiesis-stimulating agents (ESAs) (47±3.1 IU/kg), to be treated with low-molecular-weight heparin to ensure standardized anticoagulation, to be on a uniform antiplatelet regimen of aspirin at the same dose, and to have complete medical documentation.

The exclusion criteria eliminated individuals younger than 18, those on HD for fewer than six months, patients with temporary VA, and those whose initial ESA dose differed substantially because this could affect anemia and platelet-related outcomes. Also excluded were patients with malignancies, hematologic disorders, uncorrected iron deficiency, or active bleeding; patients treated with conventional heparin to avoid potential confounding from heparin-induced thrombocytopenia; individuals on steroids or immunosuppressive therapy; those who had recently received granulocyte colony-stimulating factors or blood transfusions; individuals with confirmed clinical or laboratory evidence of active infection; and patients with incomplete medical records.

A total of 156 patients were screened for eligibility. Among these, 23 patients were excluded due to incomplete clinical documentation, leaving 133 patients who met the eligibility criteria. After applying predefined exclusion criteria, 104 patients were included in the final analysis. The latter exclusions comprised four patients with temporary VA, five with substantial differences in starting ESA dose, seven with malignancies, four who had recently received blood transfusions, and nine receiving steroid therapy that could potentially influence PLTs.

The mean age of excluded patients (54.7±4.3 years) was comparable to that of the included cohort, reducing the likelihood of age-related selection bias. To further minimize selection bias, all patients who met the predefined inclusion criteria during the five-year study period were consecutively included. The exclusion process was predefined and based on objective clinical criteria, thereby limiting the possibility of subjective selection. In addition, no missing data were identified for the primary study variables, minimizing potential bias related to incomplete data.

Participants were categorized into two groups according to the type of their permanent VA: Group 1 with 51 patients with an AVF and Group 2 with 53 patients with a PC.

Variables and definitions

The primary outcome was VA dysfunction, defined as a dialysis blood flow rate (Qb) <250 mL/min, during routine HD sessions. This definition represents a functional indicator of impaired VA performance rather than direct confirmation of structural pathology such as stenosis or thrombosis. To minimize potential misclassification, patients with catheter-based VA who demonstrated reduced blood flow underwent evaluation for possible catheter malposition. Cases in which dysfunction was determined to be attributable to incorrect catheter positioning were excluded from the analysis.

The primary predictors were the mean platelet volume (MPV) and the PLT.

Secondary outcomes included dialysis adequacy indicators (single-pool Kt/V (spKt/V), urea reduction ratio (URR%)), hemoglobin levels, and weekly ESA dose. Potential confounders (ESA dose, anticoagulation type, antiplatelet therapy, inflammatory or hematologic conditions) were controlled through strict inclusion criteria and standardized protocols.

Dialysis procedure

Dialysis was performed using Fresenius 4008 and 5008 machines (Fresenius Medical Care AG & Co., Germany) under uniform prescription conditions. Low-flow polysulfone dialyzers (Etropal, Diadema (Etropal JSC, Etropole, Bulgaria) and Asahi (Asahi Kasei Medical Co., Ltd., Tokyo, Japan) brands) with surface areas of 1.8-2.1 m² were selected according to patient body surface area. All patients received approximately 12±0.30 hours of dialysis per week with bicarbonate dialysate at a flow rate of 500 mL/min. Blood flow rates (Qb) were adjusted based on VA performance and presence of dysfunction.

Laboratory measurements

Laboratory tests were performed as part of routine clinical practice in accordance with national quality control standards. Blood samples were collected following standardized procedures using the stop-pump technique, both before and/or after HD, to reduce the effects of recirculation and urea rebound.

Hgb (g/L) was measured using the colorimetric sodium lauryl sulfate method on a six-differential hematology analyzer (Sysmex XN1000, Siemens, Germany). The reference range (RR) for hemoglobin was 120-180 g/L, with a therapeutic target of 110-120 g/L for patients with end-stage renal disease (ESRD). PLT was determined using the electrical impedance method, and MPV was automatically calculated as the ratio of plateletcrit to PLT. The RR for PLT is 140-440x10⁹/L and for MPV is 6.0-10.0 fL. Blood for CBC analysis was collected into EDTA-containing tubes and analyzed within 40 minutes of sampling according to routine laboratory protocol, without interim freezing or prolonged storage.

Serum urea concentration (mmol/L) was measured by a UV kinetic method based on a coupled enzyme reaction with glutamate dehydrogenase (GLDH), performed on the ADVIA Chemistry 1800 system (Siemens, Germany). The RR was 3.2-8.2 mmol/L. Serum creatinine (mmol/L) was assessed using the kinetic Jaffe method on the same analyzer with a RR of 44-115 mmol/L.

To avoid the impact of ultrafiltration (UF), hematologic parameters, including MPV, PLT, and Hgb levels, were obtained from pre-dialysis blood samples collected according to standardized laboratory procedures. Samples for urea and creatinine were taken both pre- and post-dialysis to calculate dialysis adequacy indices, including the single-pool Kt/V (spKt/V) and the urea reduction ratio (URR).

The spKt/V was calculated using the Daugirdas second-generation equation:

spKt/V=​​-ln(R-0.008xt)+[4-3.5xR]x0.55UF/W)

where R represents the post-dialysis to pre-dialysis blood urea nitrogen ratio (C/Co), t is the dialysis duration in hours, UF denotes ultrafiltration volume in liters, W is the post-dialysis body weight in kilograms, and ln is the natural logarithm.

The URR was calculated as:

URR%=100×(1-C/Co)

where Co​ represents the pre-dialysis urea concentration and C the post-dialysis urea concentration.

Data collection

For the purposes of analysis, laboratory and dialysis-related variables were not based on single measurements. Instead, annual mean values were calculated for each patient from routinely collected clinical and laboratory data recorded during the study period. In our center, hematologic and dialysis monitoring parameters are assessed regularly, with approximately six measurements per patient per year. These averaged values were used to represent each patient’s typical clinical and dialysis characteristics and to reduce the influence of short-term biological variability. This approach allowed the longitudinal clinical records to be summarized into a single representative observation per patient for cross-sectional statistical analysis.

Bias minimization

Bias was reduced through standardized ESA, antiplatelet, and anticoagulation regimens; exclusion of patients with conditions influencing platelet indices; consistent pre-dialysis blood sampling for hematologic parameters; uniform dialysis machines and protocols; inclusion of all eligible patients to reduce selection bias; no missing data for primary variables, eliminating missing-data bias.

Residual confounding cannot be completely excluded because of the retrospective design.

Study size

A formal sample size calculation was not performed because all eligible patients during the five-year study period were included. This approach maximized sample availability and minimized sampling bias.

Statistical analysis

Statistical analysis was conducted using IBM SPSS Statistics for Windows, Version 20 (Released 2011; IBM Corp., Armonk, New York, United States). Quantitative variables were expressed as mean ± standard deviation; categorical variables as frequencies and percentages. Group comparisons were performed using Student’s t-test for normally distributed variables and Chi-square test for categorical variables. Prior to applying parametric statistical tests, the distribution of continuous variables was assessed using the Shapiro-Wilk test and visual inspection of histograms. Cohen’s d was used to estimate the effect size of between-group differences. Pearson correlation coefficients assessed linear relationships. Receiver operating characteristic (ROC) curve analysis was performed to evaluate the discriminatory performance of MPV and PLT in identifying VA dysfunction. The optimal cutoff values were determined using the Youden index, which maximizes the sum of sensitivity and specificity. The area under the ROC curve (AUC) was calculated to assess diagnostic performance. AUC values were reported with 95% confidence intervals (95% CI). Because VA type is a major determinant of access-related complications, analyses were stratified according to access type (AVF vs PC) to partially account for potential confounding. A p-value <0.05 was considered statistically significant.

Ethical considerations

The study was approved by the Research Ethics Committee of the Medical University of Varna (Protocol No. 107/28.10.2021). As data were collected retrospectively from routine clinical care, informed consent requirements were waived. A portion of the dataset was included as part of the doctoral dissertation "Adequacy of Dialysis Treatment and the Relationship with Achieved Quality of Life and Survival in Patients with Stage V Chronic Kidney Disease” of Diana Nenova (April 2022) [2].

## Results

Baseline characteristics

The baseline characteristics of the two study groups are summarized in Table 1. The groups did not differ significantly in gender distribution, prevalence of diabetes or cardiovascular disease, primary cause of ESRD, use of antihypertensive medications or aspirin, or dialysis vintage. The prevalence of VA dysfunction did not differ significantly between groups. The absence of significant baseline differences strengthens the validity of subsequent comparisons, indicating that clinical outcomes can be evaluated between the groups with minimal concern for confounding by these factors.

**Table 1:** Baseline demographic and clinical characteristics of the study groups. This table summarizes the distribution of demographic variables, comorbidities, causes of end-stage renal disease (ESRD), medication use, and dialysis vintage for Group 1 and Group 2. ESRD: end-stage renal disease; CVD: cardiovascular disease; VA: vascular access; Group 1: patients with an arteriovenous fistula (AVF); Group 2: patients with a permanent catheter (PC) * Data are expressed as number (percentage) for categorical variables and mean ± standard deviation for continuous variables, p-values are derived from the chi-square test for categorical variables, and independent samples t-tests for continuous variables.

Characteristic	Group 1 (n=51)	Group 2 (n=53)	Test statistic	p-value
Sex, Male, n (%)	23 (45.1%)	24 (45.3%)	χ²=0.000	1.000
Diabetes, n (%)	22 (43.1%)	21 (39.6%)	χ²=0.132	0.869
CVD history, n (%)	32 (62.7%)	34 (64.2%)	χ²=0.022	1.000
Cause of ESRD, n (%)				
Diabetes	28 (54.9%)	30 (56.6%)	χ²=0.03	1.000
Hypertension	15 (29.4%)	17 (32.1%)	χ²=0.09	0.935
Glomerulonephritis	8 (15.7%)	6 (11.3%)	χ²=0.44	0.715
Antihypertensives, n (%)	32 (62.7%)	30 (56.6%)	χ²=0.407	0.661
Aspirin 75 mg daily, n (%)	51 (100%)	53 (100%)	(no variability)	1.000
Dialysis vintage (years), mean ± SD	9.38 ± 2.09	9.45 ± 2.01	t=-0.174	0.862
VA dysfunction, n (%)	16 (31.4%)	19 (35.8%)	χ²=0.233	0.63

A comparative analysis of continuous baseline indicators demonstrated several significant differences between the study groups (Table 2). Although Group 2 tended to be older than Group 1 (56.77±11.28 vs. 53.06±12.07 years), the difference did not reach statistical significance (p=0.06). Measures of dialysis adequacy showed that Group 1 had significantly higher spKt/V (1.38±0.14 vs. 1.30±0.12; p=0.0009), while URR values were similar between groups (p=0.20).

**Table 2:** Data from the variational analysis and Student's t-test for the five-year period of follow-up of the two studied groups spKt/V: single-pool Kt/V, dialysis delivered dose index; URR%: urea reduction ratio, dialysis delivered dose index; Hgb (g/L): hemoglobin level; ESA (IU/week): erythropoietin stimulating agent weekly dose; MPV (fL): mean platelet volume; PLT (x10⁹/L): platelet count; Qb(mL/min): blood flow rate; Group 1: patients with an arteriovenous fistula (AVF); Group 2: patients with a permanent catheter (PC)

Indicator (X±SD)	Group 1 (n=51)	Group 2 (n=53)	t-test	p-value
Age (years)	53.06±12.07	56.77±11.28	-1.62159	0.06
spKt/V	1.38±0.14	1.30±0.12	3.20245	0.000909
URR %	68.48±3.93	67.54±7.09	0.82889	0.204552
Hgb (g/L)	110.52±4.86	104.3±5.94	5.83675	<0.00001
ESA (IU/week)	6921.57±2897.19	9358.48±2542.66	-4.5637	<0.00001
MPV (fL)	10.11±1.33	11.34±1.09	-5.20656	<0.00001
PLT (x10⁹/L)	235.74±45.23	181.83±33.68	6.91287	<0.00001
Qb(ml/min)	279.22±54.36	249.15±58.22	2.71928	0.003846

Hematologic parameters differed meaningfully. Group 1 had higher Hgb levels (110.52±4.86 vs. 104.30±5.94 g/L; p<0.00001) despite receiving significantly lower ESA doses (6921.57±2897.19 vs. 9358.48±2542.66 IU/week; p<0.00001). Platelet indices also varied: Group 1 showed lower MPV (10.11±1.33 vs. 11.34±1.09 fL; p<0.00001) but higher PLT (235.74±45.23 vs. 181.83±33.68 ×10⁹/L; p<0.00001). Additionally, blood flow rates during dialysis were significantly higher in Group 1 compared with Group 2 (279.22±54.36 vs. 256.13±56.91 mL/min; p=0.018). Overall, these findings indicate that Group 1 had more favorable hematologic parameters, higher dialysis adequacy, and required lower ESA doses compared with Group 2.

Effect size analysis using Cohen’s d was performed to provide additional context for the magnitude of between-group differences (Table 3). Large to very large effect sizes were observed for hemoglobin (Hgb; d=1.14), ESA dose (d=0.90), MPV (d=1.01), and PLT (d=1.36), indicating substantial differences in hematologic and erythropoiesis-related parameters between the groups.

**Table 3:** Effect size estimates (Cohen’s d) for differences between the study groups spKt/V: single-pool Kt/V, dialysis delivered dose index; URR%: urea reduction ratio, dialysis delivered dose index; Hgb (g/L): hemoglobin level; ESA (IU/week): erythropoietin stimulating agent weekly dose; MPV (fL): mean platelet volume; PLT (x10⁹/L): platelet count; Qb(ml/min): blood flow rate Effect sizes were interpreted according to conventional thresholds: small ≈0.2, medium ≈0.5, large ≥0.8.

Indicator	Cohen’s d	Interpretation
Age (years)	-0.32	Small
spKt/V	+0.61	Medium
URR (%)	+0.16	Small
Hgb (g/L)	+1.14	Large
ESA (IU/week)	-0.90	Large
MPV (fL)	-1.01	Large
PLT (×10⁹/L)	+1.36	Very large
Qb (mL/min)	+0.53	Medium

Moderate effect sizes were observed for dialysis adequacy and treatment parameters, including spKt/V (d=0.61) and dialysis blood flow rate (Qb; d=0.53), suggesting moderate differences in dialysis performance. In contrast, age and URR showed small effect sizes (d=-0.32 and d=0.16, respectively), indicating minimal differences between groups for these variables.

Overall, the effect size estimates provide additional context for the observed statistical differences, indicating that the main study outcomes were associated with moderate to large group differences.

**Figure 1:**
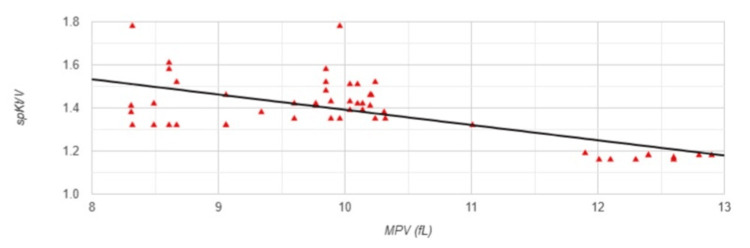
Correlation between MPV and spKt/V in Group 1 MPV: mean platelet volume; spKt/V: single-pool Kt/V, dialysis adequacy index; Group 1: patients (n=51) with an arteriovenous fistula (AVF); Pearson correlation analysis: r=-0.649, p<0.001

Pearson correlation analysis revealed several significant associations between MPV and the studied parameters in Group 1. A strong negative correlation was observed between MPV and spKt/V (r=-0.649, p<0.001) (Figure 1).

**Figure 2:**
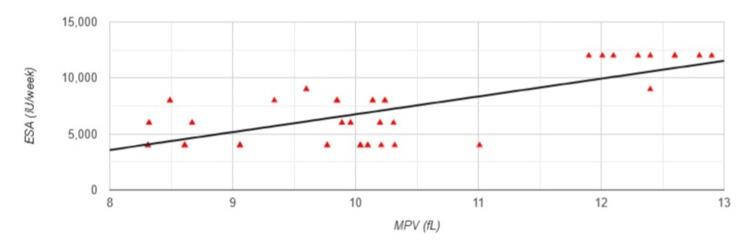
Correlation between MPV and ESA in Group 1 MPV: mean platelet volume; ESA: erythropoiesis-stimulating agent; Group 1: patients (n=51) with an arteriovenous fistula (AVF); Pearson correlation analysis: r=0.731, p<0.001

**Figure 3:**
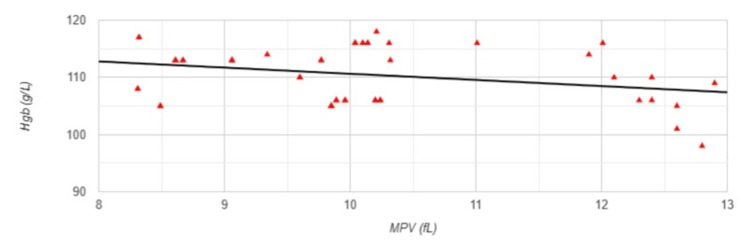
Correlation between MPV and Hgb levels in Group 1 MPV: mean platelet volume; Hgb: hemoglobin levels; Group 1: patients (n=51) with an arteriovenous fistula (AVF); Pearson correlation analysis: r=-0.295, p=0.036

Regarding anemia-related parameters, a strong positive correlation was observed between MPV and the administered erythropoiesis-stimulating agent (ESA) dose (r=0.731, p<0.001) (Figure 2). Conversely, a weak but statistically significant negative correlation was observed between MPV and hemoglobin levels (r=-0.295, p=0.036) (Figure 3).

**Figure 4:**
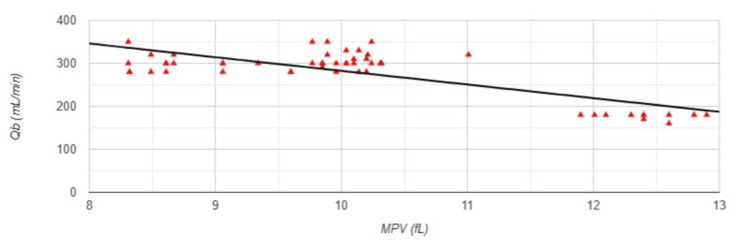
Correlation between MPV and blood flow rate in Group 1 MPV: mean platelet volume; Qb: blood flow rate; Group 1: patients (n=51) with an arteriovenous fistula (AVF); Pearson correlation analysis: r=-0.777, p<0.001

A strong negative correlation was observed between MPV and Qb (r=-0.777, p<0.001) (Figure 4).

**Figure 5:**
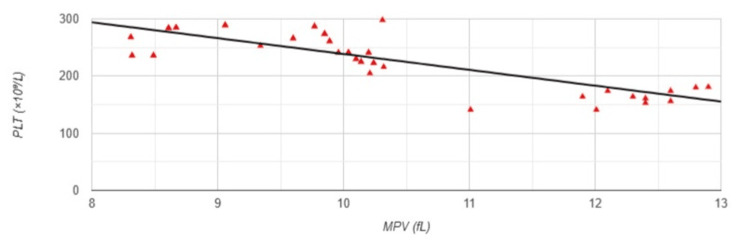
Correlation between MPV and PLT in Group 1 MPV: mean platelet volume; PLT: platelet count; Group 1: patients (n=51) with an arteriovenous fistula (AVF); Pearson correlation analysis: r=-0.758, p<0.001

Pearson correlation analysis revealed a strong negative correlation between MPV and PLT (r=-0.758, p<0.001) (Figure 5).

**Figure 6:**
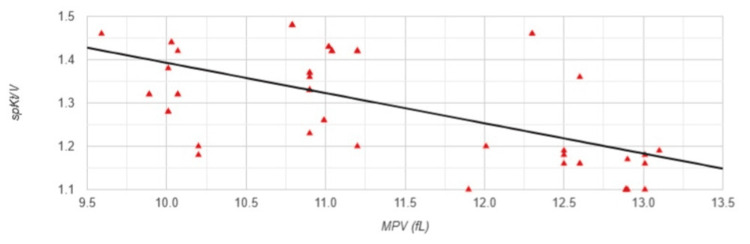
Correlation between MPV and spKt/V in Group 2 MPV: mean platelet volume; spKt/V: single-pool Kt/V, dialysis adequacy index; Group 2: patients (n=53) with a permanent catheter (PC); Pearson correlation analysis: r=-0.605, p<0.001

The same types of correlations between MPV and the studied parameters were observed in Group 2. A strong negative correlation was found between MPV and spKt/V (r=-0.605, p<0.001) (Figure 6).

**Figure 7:**
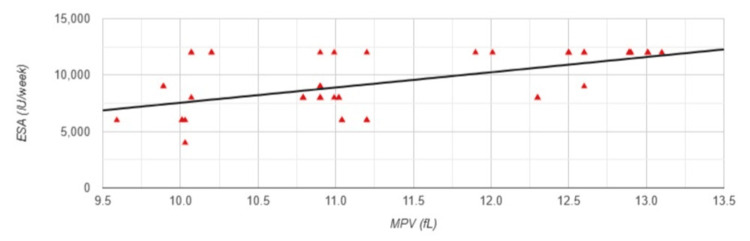
Correlation between MPV and ESA in Group 2 MPV: mean platelet volume; ESA: erythropoiesis-stimulating agent; Group 2: patients (n=53) with a permanent catheter (PC); Pearson correlation analysis: r=0.578, p<0.001

**Figure 8:**
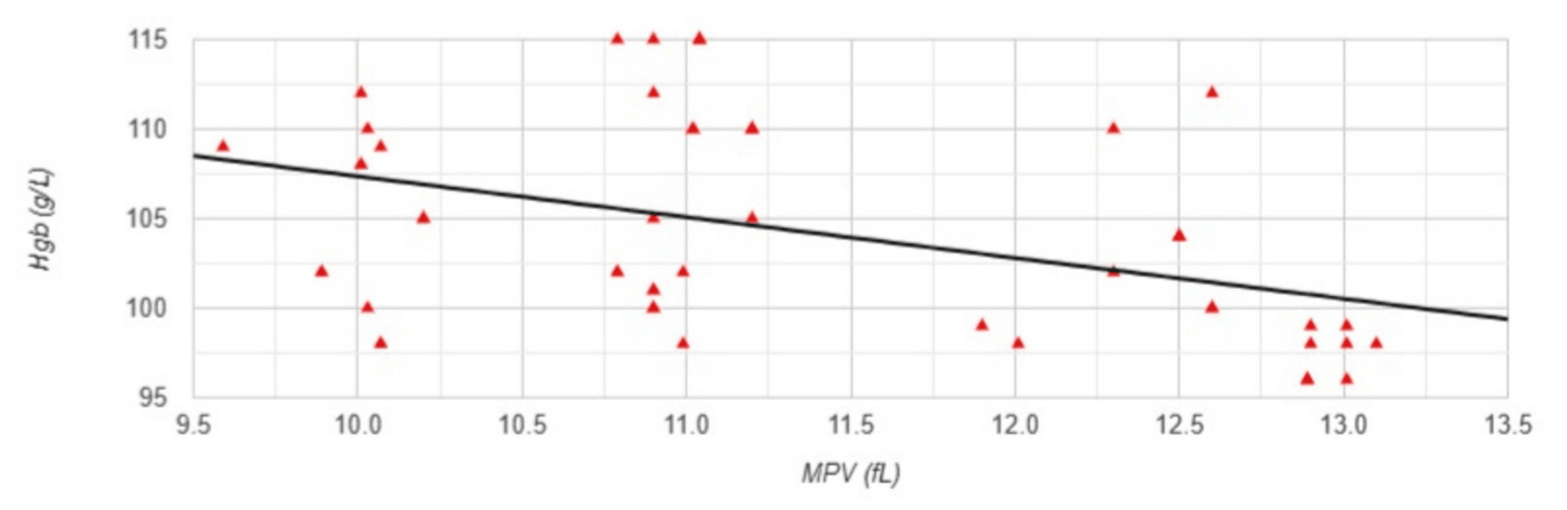
Correlation between MPV and hemoglobin levels in Group 2 MPV: mean platelet volume; Hgb: hemoglobin levels; Group 2: patients (n=53) with a permanent catheter (PC); Pearson correlation analysis: r=-0.417, p=0.002

The results show that there is a significantly large positive correlation between MPV and ESA (r=0.578, p<0.001) (Figure 7) and significant medium negative correlation between MPV and Hgb levels (r=-0.417, p=0.002) (Figure 8).

**Figure 9:**
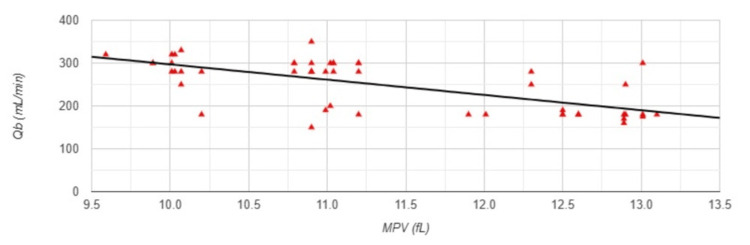
Correlation between MPV and blood flow rate in Group 2 MPV: mean platelet volume; Qb: blood flow rate; Group 2: patients (n=53) with a permanent catheter (PC); Pearson correlation analysis: r=-0.666, p<0.001

Regarding estimated blood flow rate, there was a significantly large negative correlation between MPV and Qb (r=-0.666, p<0.001) (Figure 9).

**Figure 10:**
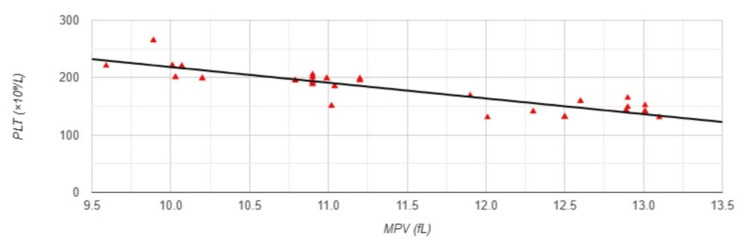
Correlation between MPV and PLT in Group 2 MPV: mean platelet volume; PLT: platelet count; Group 2: patients (n=53) with a permanent catheter (PC); Pearson correlation analysis: r=-0.883, p<0.001

A significant large negative association between MPV and PLT, (r=-0.883, p<0.001) was also found in Group 2 (Figure 10).

**Figure 11:**
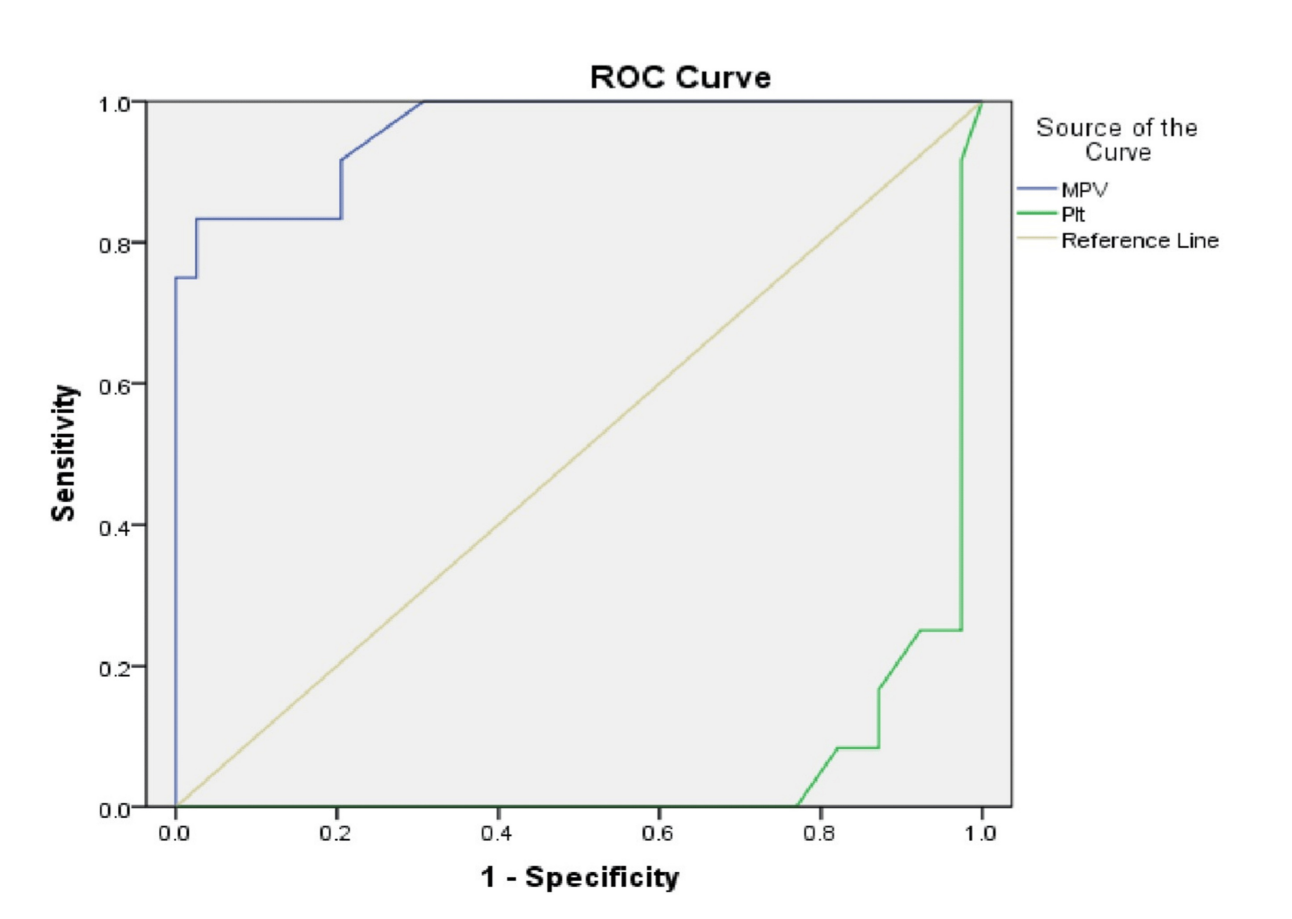
ROC curves evaluating the discriminatory performance of MPV and PLT for identifying vascular access dysfunction in Group 1 with an AUC of 0.96 (95% CI: 0.904-1.00, p<0.0001). ROC: receiver operating characteristic; blue line: mean platelet volume (MPV); green line: platelet count (PLT); yellow line: reference line; AUC: area under curve; Group 1: patients (n=51) with an arteriovenous fistula (AVF)

**Figure 12:**
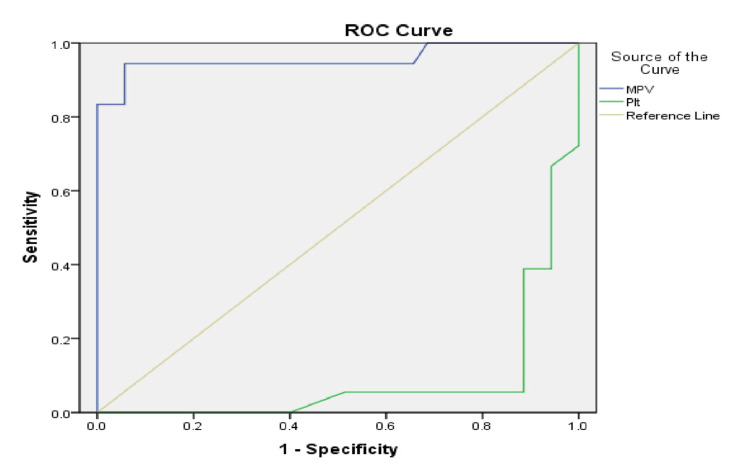
ROC curves evaluating the discriminatory performance of MPV and PLT for identifying vascular access dysfunction in Group 2 with an AUC of 0.956 (95% CI: 0.884-1.00; p<0.001). ROC: receiver operating characteristic; blue line: mean platelet volume (MPV); green line: platelet count (PLT); yellow line: reference line; AUC: area under the curve; Group 2: patients (n=53) with a permanent catheter (PC)

Figure 11 and Figure 12 present the ROC curves evaluating the discriminatory performance of MPV and PLT in identifying VA dysfunction in both groups across the five-year study period. The ROC analyses identified group-specific cut-off values.

ROC analysis in the AVF group demonstrated that MPV had strong discriminatory performance for identifying VA dysfunction, with an AUC exceeding 0.9. Specifically, MPV showed an AUC of 0.96 (95% CI: 0.904-1.00; p<0.0001). The optimal cut-off value determined by the Youden index was MPV>10.27 fL, corresponding to a sensitivity of 83.3% and specificity of 84.6%.

In contrast, PLT demonstrated a statistically significant inverse discriminatory pattern (AUC=0.053; 95% CI: 0.010-0.096; p<0.001), indicating that lower PLT values were associated with VA dysfunction.

In the PC group, ROC analysis also demonstrated strong discriminatory performance of MPV (AUC=0.956, 95% CI: 0.884-1.00; p<0.001). The optimal threshold identified by the Youden index was MPV>11.5 fL, corresponding to a sensitivity of 94.4% and specificity of 94.3%. PLT likewise showed a statistically significant inverse discriminatory pattern (AUC=0.086, 95% CI: 0.05-0.167; p<0.001), indicating that lower PLT values were associated with VA dysfunction; however, no clinically practical cutoff value could be identified.

Overall, MPV demonstrated clearer discriminatory characteristics and a well-defined threshold, suggesting potential clinical usefulness compared with PLT.

## Discussion

The present study suggests that MPV is strongly associated with VA dysfunction in patients undergoing chronic HD and may represent a more clinically informative marker than PLT. Elevated MPV values were consistently associated with impaired VA performance, reduced dialysis adequacy, and poorer anemia control, and these associations were observed in both AVF and PC patient groups. Although PLT also showed a statistically significant association with VA dysfunction, its inverse discriminatory pattern and the lack of a clinically meaningful threshold limit its practical interpretability. In contrast, MPV demonstrated clearer discriminatory characteristics with identifiable cutoff values, suggesting potential utility as a marker reflecting platelet activation and biological processes that may influence VA performance and HD outcomes.

ROC analysis further demonstrated good discriminatory performance of MPV for identifying VA dysfunction. In the AVF group, MPV showed a high area under the curve (AUC=0.96), while in the catheter group, the AUC remained similarly elevated (AUC=0.956), indicating strong discriminatory ability across both VA types. The corresponding optimal cutoff values, approximately 10.27 fL in AVF patients and above 11.5 fL in catheter users, were associated with favorable sensitivity and specificity values, suggesting potential clinical usefulness of MPV as a threshold-based marker.

In contrast, PLT demonstrated a statistically significant inverse discriminatory pattern, with AUC values close to zero in both groups. This finding suggests that lower PLTs were associated with impaired VA function, rather than indicating an absence of discrimination. Nevertheless, the inverse direction of this association and the absence of a clinically useful cutoff limit the practical diagnostic utility of PLT.

Together, these findings suggest that markers reflecting platelet activation and platelet size, such as MPV, may provide more clinically interpretable information regarding VA dysfunction than platelet quantity alone, possibly reflecting underlying pathophysiological mechanisms involved in access failure [15,16].

These findings are consistent with accumulating evidence from other centers. Previous studies, including prospective analyses such as that of Lano et al. (2019), have reported that higher MPV is associated with an increased risk of VA events. Similarly, in our cohort, elevated MPV values were associated with higher rates of access dysfunction across both VA types (AVF and PC) [15]. These observations are compatible with the pathophysiological concept that larger, more metabolically active platelets exhibit increased reactivity and may contribute to thrombotic or stenotic processes affecting VA function. The inverse discriminatory pattern observed for PLT in ROC analysis further suggests that platelet quantity alone may be less informative than indices reflecting platelet size and activation. Although AVF demonstrated superior clinical performance, including higher blood flow, improved anemia control, and better dialysis adequacy, the association between MPV and VA dysfunction was observed across both access modalities.

From a mechanistic perspective, chronic kidney disease and uremia are associated with pro-thrombotic and pro-inflammatory states, endothelial dysfunction, and altered vascular hemodynamics in AVFs or catheters, conditions that may promote platelet activation and vascular remodeling [23].

Correlation analyses in our study further support the potential relevance of platelet activation in HD patients. The observed negative correlation between MPV and dialysis adequacy indices (spKt/V) in both groups suggests that increased platelet reactivity may be associated with hemodynamic changes or thrombotic tendencies that limit effective blood flow during dialysis. Similar observations have been reported in previous studies, where elevated MPV or MPV/PLT ratios were linked to VA complications as well as markers of inflammation, oxidative stress, and erythropoiesis resistance [15,16,24]. In addition, the negative association between MPV and dialysis blood flow (Qb) observed in our analysis may reflect impaired VA function contributing to reduced flow rates and subsequent limitations in dialysis adequacy. Given that suboptimal Qb is a key determinant of insufficient solute clearance, these findings may have relevant clinical implications [15,25-28].

A notable finding is the strong association between MPV and the required dose of erythropoietin. As a hematopoietic growth factor, erythropoietin can enhance platelet activation, and higher doses used to achieve target Hgb levels may promote thrombogenesis. Managing anemia in HD patients is frequently challenging due to inflammation, impaired iron utilization, and repeated blood loss or hemolysis associated with VA. Increased platelet activity may signal underlying vascular or inflammatory stress, which in turn can contribute to greater ESA needs and reduced Hgb levels [29]. In this context, MPV may represent a parameter linking VA function, dialysis adequacy, and anemia management in HD patients.

An important finding of the present study is the strong and consistent inverse correlation between MPV and PLT observed in both VA groups (AVF: r=-0.758; PC: r=-0.883), indicating that increases in platelet size were accompanied by reductions in PLT. This relationship is well recognized in hematological physiology, where MPV and PLT demonstrate a natural inverse regulatory balance driven by thrombopoietic feedback mechanisms: larger, younger, and more reactive platelets are typically produced in states of increased platelet turnover or activation, resulting in a relative decrease in total platelet number [17]. In HD patients, chronic endothelial injury, inflammation, and exposure of platelets to bioincompatible surfaces contribute to accelerated platelet consumption and activation, which are reflected by higher MPV values despite lower PLT counts. This pattern has been reported previously in dialysis cohorts, where elevated MPV or elevated MPV/PLT ratios were associated with thrombosis, inflammation, and AVF failure [15,24].

In the present study, the stronger inverse MPV-PLT correlation in the catheter group may reflect a heightened pro-thrombotic and inflammatory environment associated with permanent central venous catheters compared with AVFs. Catheters are known to induce persistent endothelial irritation, turbulent flow, and a higher degree of systemic inflammatory activation, all of which can enhance platelet turnover and activation. The combined effect of reduced PLT and increased platelet reactivity (indicated by higher MPV) may therefore contribute to the markedly impaired blood flow (Qb) and greater access dysfunction observed in this group. These findings are consistent with previous reports suggesting that MPV may reflect platelet activation more effectively than PLT alone and may represent an indirect indicator of vascular stress, endothelial injury, and thrombotic tendency in HD patients [18,19].

Overall, the observed MPV-PLT relationship provides additional pathophysiological context for the association between platelet indices and VA performance. The consistent inverse correlation observed across both access groups suggests that MPV may capture aspects of platelet activation dynamics that are not reflected by PLT alone. However, given the observational design of the present study, these findings should be interpreted cautiously. Further prospective studies are needed to determine whether longitudinal monitoring of MPV or MPV/PLT trends could assist in identifying patients at increased risk of VA dysfunction.

Clinical implications and utility

Given that MPV is routinely reported as part of standard complete blood count panels, its potential use as a biomarker for VA risk stratification is attractive, as it requires no additional cost or specialized testing [15]. Elevated MPV values may help identify patients who could benefit from closer monitoring of VA function, such as duplex ultrasound evaluation or access flow measurements, with the aim of detecting early signs of dysfunction. Current European and international guidelines recommend regular and objective monitoring of access function (ideally every month for arteriovenous graft and every three months for AVF) [30]. Incorporating readily available laboratory parameters such as MPV into clinical assessment may be particularly useful in settings where resource constraints limit routine in-depth vascular surveillance [14,30,31].

In addition, MPV may help identify patients at increased risk of suboptimal dialysis delivery or inadequate anemia control related to VA dysfunction, potentially allowing earlier clinical adjustments in dialysis prescription or ESA dosing.

Strengths and limitations

An important strength of this study is the careful control of several potential confounding factors, including standardized ESA dosing, a uniform heparin protocol, consistent antiplatelet therapy, and the exclusion of patients with malignancy, infection, active bleeding, or recent blood transfusion. These methodological precautions improve internal consistency and reduce the influence of factors known to affect platelet indices, allowing a clearer evaluation of the association between platelet parameters and VA performance. Although analyses were stratified by VA type, residual confounding from other clinical variables cannot be excluded.

Several limitations of this study should be acknowledged. First, the retrospective single-center design limits the ability to establish causal relationships and may restrict the generalizability of the findings to other HD populations. Second, although strict inclusion and exclusion criteria were applied to reduce major confounding factors, residual confounding cannot be completely excluded, particularly given the absence of extensive multivariable adjustment and the potential influence of factors such as inflammation, nutritional status, and overall comorbidity burden. Third, VA dysfunction was defined using a functional criterion (inability to maintain dialysis blood flow rate ≥250 mL/min), which represents a pragmatic indicator of impaired access performance but does not necessarily confirm structural abnormalities such as stenosis or thrombosis. Fourth, the study population was relatively selected, including patients receiving uniform aspirin therapy and comparable baseline ESA dosing, which may reduce heterogeneity but could also limit external validity. In addition, the study focused exclusively on patients undergoing chronic HD with established VA; therefore, the findings may not be generalizable to patients receiving peritoneal dialysis or those with newly created VA. Furthermore, MPV is known to be influenced by pre-analytical factors, including timing of analysis and sample handling, although standardized laboratory procedures were used in our center to minimize variability. Finally, the modest sample size warrants cautious interpretation of the results, and larger prospective multicenter studies are needed to confirm the observed associations and further evaluate the potential clinical relevance of platelet indices in relation to VA dysfunction.

Despite these limitations, the present findings provide preliminary evidence supporting the potential clinical relevance of MPV as a readily available parameter associated with VA performance in patients undergoing chronic HD. However, platelet function is complex, and MPV represents only a surrogate marker. More direct assessments of platelet activation, such as flow cytometry-based markers, platelet aggregation assays, or shear-dependent adhesion testing, may provide additional mechanistic insight but are not always feasible in routine clinical practice.

## Conclusions

In conclusion, the present study suggests that MPV, a routinely available hematological parameter, is associated with VA performance in patients undergoing chronic HD. Given its low cost and widespread availability as part of standard complete blood count testing, MPV may represent a clinically accessible marker that could help identify patients at higher risk of VA dysfunction. However, due to the retrospective observational design of this study, the findings should be interpreted cautiously. Prospective, multicenter studies are needed to validate these associations and to determine whether MPV-based risk assessment could contribute to improved monitoring and management of VA in HD patients.
